# Pattern, Management, and Outcomes of Chest Injury At Kilimanjaro Christian Medical Centre

**DOI:** 10.24248/eahrj.v7i1.714

**Published:** 2023-07-12

**Authors:** Elias Mduma, Samwel Chugulu, David Msuya, Francis Sakita, L,ele Mutombo Fabrice

**Affiliations:** a General Surgery, Kilimanjaro Christian Medical Centre; b Emergency Medicine Department, Kilimanjaro Christian Medical Centre; c Kilimanjaro Christian Medical University College

## Abstract

**Background:**

Chest trauma is a major cause of morbidity and mortality in the region. Lacking data in our environment has been a challenging part of knowing the burden of the problem. Long hospital stays and associated injuries are an essential measure of morbidity. The study results will provide a basis for planning prevention strategies and establishment of treatment protocols.

**Objectives:**

To determine the prevalence, pattern, and management outcomes of chest injury patients at Kilimanjaro Christian Medical Center (KCMC), a Tertiary Hospital in Northern zone Tanzania from October 2021 to April 2022.

**Methodology:**

A hospital-based cross-sectional study was conducted among patients with chest injuries who were admitted and managed at Tertiary Hospital Northern Zone (Kilimanjaro Christian Medical Center-KCMC) in the Emergency medicine and General Surgery departments. Using a designated data collection tool, details of the mechanism of injury, radiological and laboratory investigations, management, and outcomes were recorded.

**Results:**

A total of 114 chest injury patients were studied. Males outnumbered females by a ratio of 7.14:1. Their ages ranged from 2 to 83 years (mean = 36.18 years). The Majority of patients (95.58%) sustained blunt injuries. Road traffic crush was the most common cause of injuries affecting 65.79% of patients. Lung contusion, hemothorax, and rib fractures were the most common type of injuries accounting for 54.4%, 27.2%, and 21.1%, respectively. Associated injuries were noted in 85.7% of patients, and head injury (60.5%) was found in most patients. The Majority of patients (60.5%) were treated successfully with a non-operative approach. Underwater seal drainage was performed at (38.9%). One Patient (0.9%) underwent a thoracotomy. 14% of patients had complications of surgical site infection, and 69% were found in the Majority of patients. The median length of hospital stay was 4.5 days. The mortality rate was 21%

**Conclusion:**

Motor traffic crash was the principal cause of chest trauma. Young male patients were most affected by chest trauma and the majority of patients were treated conservatively. Chest X-ray remains to be the main imaging modality for diagnosing thoracic trauma lesions. Associated injuries such as head injuries, were found to contribute to a high mortality rate.

## BACKGROUND

Injury is a major public health problem in both developed and developing countries and is responsible for about 5.8 million deaths per year worldwide^1^. Road traffic crash (RTC), is the leading cause of traumatic deaths, and they are responsible for approximately more than 2.7 million injuries related per annum. Out of this, 91% of injury-related deaths occurred in developing countries.^2^

Chest trauma is responsible for 10% of all trauma admissions and 25% of trauma-related death globally.^3^ Several studies in Africa showed that chest trauma is a major cause of morbidity and mortality in the region.^4^ In Tanzania, it has been reported to be one of the leading causes of morbidity among polytrauma patients.^4^

The pattern of chest injuries varies widely and essentially depends on the environment or the kinematics and severity of the accidents in the diverse geographical regions worldwide.^2^ The commonest cause of blunt chest injury has been reported to be road traffic crashes, although the incidence of penetrating chest injuries has also increased in civil society due to increasing the use of firearms, and other traditional weapons.^5^ Associated injuries play an important role in determining the outcome of a chest injury patient especially when a head injury is associated.^6^

The management has several essential elements, such as adequate prehospital care, rapid transport to a specialized center, complex in-hospital care, and rehabilitation. The prehospital phase is vital in determining treatment outcomes appropriately and contributes significantly to reducing morbidity and mortality.^7^ Few thoracic trauma patients require a surgical operation, and a majority of patients can be treated with simple methods such as appropriate airway maneuvers, oxygen support, fluid therapy, and tube thoracostomy.^8^

The mortality rate of patients may not entirely result from the distortion of chest wall architecture and abnormal mechanics of breathing but may also result from bilateral impact and pulmonary contusion translating to a worsened degree of hypoxemia.^9^ Long hospital stay has been a major problem in patients with penetrating chest injuries and those with associated extra thoracic injuries and is an essential measure of morbidity.^4^Lacking data in our environment has been a challenging part of knowing the burden of the problem. This study aimed to know the burden of chest injury-related disorders in our region so that to describe our own experience in the management of chest injuries, outlining the cause spectrum, injury patterns, and outcome in the management of chest injuries in our local setting. The study results provides a basis for developing prevention strategies and establishment of treatment protocols.

## PATIENTS AND METHODS

### Study design and Study site

A hospital-based descriptive cross-sectional study was conducted at the Emergency Medicine Department and General Surgery wards in Kilimanjaro Christian Medical Center, from October 2021 to March 2022. The facility also serves as a teaching hospital for the Kilimanjaro Christian Medical University College (KCMUco).

### Sample Size and Sampling Technique

The sample size was estimated by the Kish Leslie formula, (1965) for cross-sectional studies. Thus, considering the proportion of chest trauma of 6% (Dogrul et al.,2020), assuming a confidence level of 95% and a margin of error of 5%, the calculated sample size was 86. Accounting for a non-response of 10%, the sample size was increased to 95. C

### Study Participants

All individuals with chest trauma who were admitted, managed, and discharged during the study period.

### Data Collection and Analysis

Data was collected using a well-structured electronic questionnaire The collected information included demographic profile, mode of injury, types of chest injuries, management, and outcome.

Descriptive analysis was performed using EPI-INFO 7 (Version 7.2.5.0 of March 2022). A chi-square (χ2) test was performed to compare experience of chest trauma between groups. Multivariate logistic regression analysis was used to determine predictor variables that are associated with outcome. A P-value of less than 0.05 was considered statistically significant.

### Ethical Consideration

Ethical approval to conduct the study was obtained from the Research and Ethics Committee of KCMUCo, Tumaini University Makumira(Reference Number PG89/2022). Informed consent was sought from each patient before recruitment into the study. And for those under 18 years of age; when the child was able to understand the study both his assent and the parent's or/and caretaker's consent were sought. For young children, unable to understand the study, the consent of the parent or/and caretaker was sufficient to be enrolled. The study observed the confidentiality and privacy of the subjects

## RESULTS

Our study enrolled 588 traumatized patients admitted to the EMD of KCMC, of which 114 cases (19.4%.) were thoracic trauma. The mean age of 114 patients with chest trauma was 36 ± 16 (range from 2 years to 83 years) and 72.8% belonged to the age group of 20–60 years. The male-to-female ratio was 7: 1 and the majority of the patients were not insured (93%) and 86.84% had no previous history of injury (Table 1)

**TABLE 1:** Characteristics of Study Participants

Characteristic	Male (%)	Female (%)	Total (%)
**Age**			
1-19	9 (7.89)	4 (3.51)	13 (11.40)
20-39	51 (35.41)	4 (3.51)	55 (38.19)
40–59	32 (22.22)	4 (3.51)	36 (25)
60-79	7 (6.14)	2 (1.39)	9 (7.89)
80–99	1 (0.69)	0 (0.00)	1 (0.69)
Total	100 (69.44)	14 (9.72)	114 (100)
Mean age	36.16 ± 16.69 years		
**Insured**			
Yes	6 (5.26)	2 (1.39)	8 (7.02)
No	94 (82.46)	12 (10.53)	106 (92.98)
**Toxic habit and medical history**			
Smoking	0 (0.00)	12 (10.53)	12 (10.53)
Alcoholic	72 (63.16)	5 (4.38)	77 (67.54)
Surgical history	1 (0.69)	10 (8.77)	11 (9.64)
Hypertension	0 (0.00)	9 (7.89)	9 (7.89)
Diabetes	0 (0.00)	1 (0.69)	1 (0.69)
**Type of Trauma**			
Blunt	14 (9.72)	94 (82.46)	108 (94.73)
Penetrating	0 (0.00)	6 (5.26)	6 (5.26)

The most common mode of injury was motor traffic crashes (MTC) in 75 (65.79 %) patients. Among patients of MTC, 28.1% of cases were involved in motorized two or three-wheeler crashes, 16.7% of patients had four-wheeler-related injuries, and the remaining 21%were pedestrians hit by moving vehicles(Table 2).

**TABLE 2:** Cause of Trauma

Variable	Frequency	Percent (%)
**MTC (total)**	**75**	**65.79**
*- Car driver*	*2*	*1.4*
*- Motorcyclist/BAJAJ*	*32*	*28.1*
*- Passenger on board a vehicle*	*17*	*14.9*
*- Pedestrian*	*24*	*21.1*
**Accident at work**	**5**	**4.4**
**Sports accident**	**1**	**0.9**
**Fall**	**13**	**20.3**
**Assault**	**10**	**8.9**
*- Animal attack*	*1*	*0.9*
*- Blunt weapon*	*5*	*4.4*
*- Sharp Object*	*4*	*3.5*

The majority (96.5%) of the individuals who experienced thoracic trauma were involved in severe accidents, of which 26.5% were seen at the hospital during the six first hours post-accident, and 42.5% were admitted 24 hours post the accident. Isolated chest injuries were present in 16 (14.3%) patients and the remaining 98 (85.7%) patients were diagnosed as polytrauma cases. Even if 83.2% of patients used an ambulance to go to the hospital, no adequate resuscitation was performed, no IV line and no IV fluid was given (Table 3).

**TABLE 3:** Severity, Type and Associated Injuries of Chest Injury

Characteristic	Frequency	Percent
**Time between trauma and admission**		
- Less than 6 hours	30	26.6%
- 6 hours to 24 hours	35	30.9%
- More than 24 hours	49	42.5%
**Transport**		
- By the family/Good Samaritan	19	16.8%
- Medicalized (ambulance)	04	83.2%
**Severity of the accident**		
- Yes	110	96.5%
**Type of lesions**		
- Isolated Chest trauma	16	14.3%
- Polytrauma	08	85.7%
**Associated injury in polytrauma**		
- Neck	2	1.8%
- Head	69	60.5%
- Lumbar spine	2	21.15%
- Abdominal 10 8.77%	10	8.8%
- Pelvic	55	48.2%
- Limbs	55	48.2%

**FIGURE 1:**
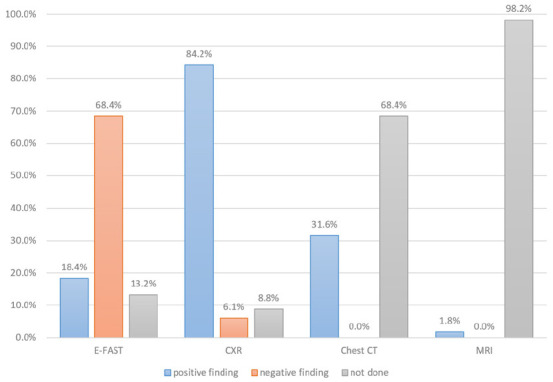
Types of Imaging Tools Used to Diagnose Chest Injury

The most used imaging modality was the e-FAST (114 patients) followed by a chest X-ray (113 patients). The chest X-rays showed positive findings in 84.9% of cases and negative findings in 6.2% of cases. While e-FAST showed positive findings in 18.42% and negative findings in 68.42%. Chest X-ray was more accurate in diagnosing traumatic chest injury than e-FAST(Figure 1).

**FIGURE 2:**
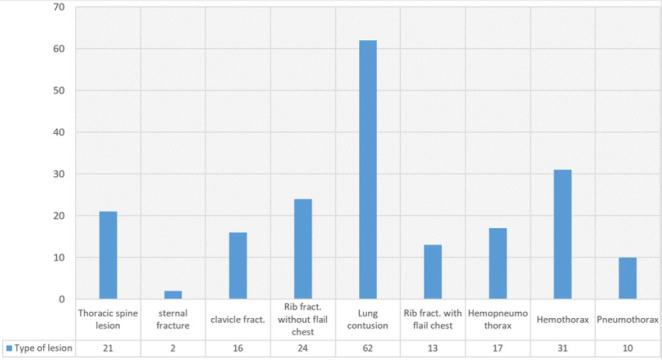
Type of Lesion

At presentation, lung contusion 54.4% was the most frequent injury, either as an isolated injury or associated with other thoracic or extrathoracic injuries. The following common injuries were rib fractures with or without flail chest and hemothorax 27.2% (Figure 2).

**FIGURE 3:**
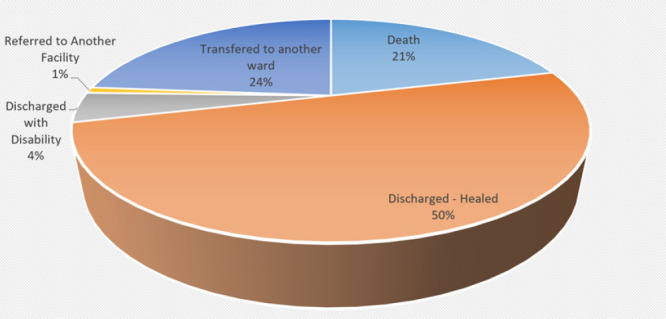
Outcome

Conservative management was performed in 40.4% of patients and the most common surgical procedure was chest tube insertion, carried out in 44 (38.9%) patients of which 79.2% was unilateral tube insertion and 20.9% bilateral. The mean duration of the chest tube was 6.8 ± 3.9 days with a median of 6 days (range 1–20 days). The chest tube was inserted in 53.5% of patients for hemothorax, 34.9% for hemopneumothorax, and 11.6% for pneumothorax (Table 4).

**TABLE 4:** Management of Chest Injury

Characteristic	Frequency	Percent (%)
**Urgent Management**		
Transfusion	34	29.8
Vasopressor drugs	21	18.4
Intubation	23	20.2
Analgesics	114	100.0
Antibiotic	109	96.5
Fluid	108	94.7
Oxygen support (nasal/facial mask)	63	55.3
**Management of chest trauma**		
Chest drainage	44	38.9
Mean duration of drainage	6.78 days ± 3.95 days	
Conservative Management	69	60.5
Thoracotomy	1	0.9
**Treatment of associated injury**		
Surgical debridement	9	7.9
Laparotomy	9	7.9
Assisted ventilation	19	16.7
Burr hole/Craniotomy	5	4.4
Open/closed fracture reduction	55	48.5

Mortality rates did not vary between blunt and penetrating injury groups. The majority of patients (66. 7%) were hospitalized for less than one week. The median length of hospital stay was 4.5 days, ranging from 1 to 49 days. A chest trauma patient with a severe TBI component is 12 times more likely to die. (Table 5).

**TABLE 5:** Multivariate Logistic Regression Analysis of Outcome Among Chest Trauma Patients

OUTCOME			OR	Sig.	95% Confidence	
					Lower Bound	Upper Bound
DEATH	GCS	***SEVERE TBI***	***12.4***	***.006***	***2.058***	***74.659***
		MODERATE TBI	1.2	.866	.267	4.798
		MILD TBI		.	.	.
	RESP RATE	BRADYPNE A	2.2	.845	.026	86.570
		TACHYPNE A	2.1	.620	.191	16.106
		EUPNEA		.	.	.
	HEAD INJURY	YES	2.1	.131	.731	11.186
		NO	.	.	.	.

The Reference Category is: ALIVE

## DISCUSSION

The proportion of traumatized patients with chest injuries was 19.4% which is lower than the proportions reported in other studies.^10–12^ ^101112^ The difference can be explained by the fact that our study essentially took place during the rainy season. season in which the motorcycle, the main means of transport for the population and the main contributor to motor traffic crash (MTC) cases, is underused. In line with the findings of other studies,^10–12^ most of the patients were males aged 20 to 40 years. Young males are involved in high-risk-taking daily activities and outdoor activities like driving and other hazardous occupations. Similar findings were obtained from studies done by Lema et al who noticed a predominance of the male sex (sex ratio 3.8:1) and 61% of patients aged 21-40.^4^

The most common mode of injury was motor traffic crash (MTC) involving motorized two or three-wheeler crashes and minority of patients had four-wheeler-related injuries, the remaining victims were pedestrians hit by moving vehicles. This finding was consistent with the results of studies conducted in Tanzania.^4,13^ Motorcyclists were mostly affected due to their occupational exposure. Challenges of infrastructures, overcrowded by pedestrians and petty traders made them to be in a high risk of being knocked by moving vehicles.

The majority of patients in this study arrived in the hospital 24 hours post-injury, similar to the study done by Lema et al.^4^, and Baru et al.^14^, which showed the same results. The late presentation is due to a delayed decision in seeking medical attention after injury, and once the decision is made, patients first pass through lower health centers before being referred to the higher specialized centers. Knowing the time of injury in trauma patients is essential for prevention strategies and has an impact on the outcome. Baru et al^14^ in Ethiopia reported that one-third of the victims reached healthcare facilities within a golden hour and had good outcomes, while the late presentation was highly associated with bad outcomes.

In this study, a significant number of patients were declared severely injured due to a high mechanism of injury, in contrast to the study done by Lema et al.^4^, where the majority of patients were not severely injured. Despite the fact of patients were transported by ambulance, there was inadequate resuscitation of the patients as a majority were brought unstable. Blunt injuries represent the majority of chest trauma patients encountered. The findings are similar to other studies conducted in different parts of the world.^4,15–17^ In this study, most of the patients suffered motor traffic crush with direct injury to the chest. However, in Nigeria, the^18^ majority of patients experienced a penetrating injury that was due to the high rate of crime that existed in that region. In our area, a small number of penetrated injuries were due to violence and assaults.

Similar to the findings reported by Huber et al,^10^ lung contusion was found in a majority of a patient as an isolated injury or associated with other thoracic or extrathoracic injuries. But these findings were different from what has been reported in studies conducted in the same geographical locations,^4,13,17^ which reported different types of chest injury such as chest wall wounds, and rib fractures. Most lesions occurred on the right side, probably due to the dominancy of the right side by many patients, and during the mechanism of injury, people tend to resist by using the strongest side of the body parts.

The presence of associated injuries was found in the majority of the patients, predominantly head injuries. Similarly, Lema et al^4^ reported that head injury tends to be a significant associated injury and it plays an important role in determining the outcome of a chest injury patient.^13,18^ Chest X-ray has been reported to be an important diagnostic tool in the diagnosis of Lung contusion, rib fractures, and hemopneumothorax.^8^ However, another study^4^ acknowledged that ultrasound is the best diagnostic tool for hemopneumothorax. Like in other studies,^18^ chest radiography was done on all patients.^18^ The current study found that 60.5% of patients were managed conservatively, and 39.8% were managed with surgical interventions such as chest tube thoracostomy and thoracotomy (38.9% and 0.9%, respectively). The findings were similar to those reported by Lema et al^4^ but in contrast to those reported by Massaga et al,^13^ where a majority of patients were treated by surgical intervention. The chest tube was inserted in the Majority (53.5%) of patients with hemothorax and 34.9% for hemopneumothorax, and the findings were almost similar to other studies.^11,18^ The mortality rate was 21.1%, which was high compared to the rates reported by other studies conducted in the country.^4,13^. The majority of patients in this study experienced a severe form of injuries, chest injury associated with other injuries, and presented at the hospital 24 hours post-injury. Associated injuries, particularly head injuries, impacted negatively the mortality rate. This is in line with the findings of other studies.^17^ The instability of the patient and respiratory rates were predictors of poor outcomes.

The current study found a complication rate of 14% of all managed chest trauma patients which is lower than the rates reported by other studies.^13,17^ While the current study revealed that surgical site infection/wound infection was the common complication, pneumonia^17^ and nonfunctional tubes^13^ were reported by other studies^1713^. The median length of hospital stay was 4.5 days, different from other studies which reported a median of more than 5 days.^4,13^The GCS less than 8 was positively associated with the death. For lema, Masuma and Lema report also, respectively, a positive association of Associated injuries with death in patient with cest trauma.^19, 4^

## CONCLUSION

Chest injury is common in motor traffic accidents seen at KCMC. Commonly affected victims are young adult males in their productive and reproductive age group. However, most of the individuals who sustain injuries are first kept in a peripheral center with inadequate treatement and then present at KCMC 24 hours post-injury. Associated head injury increased the risk of poor outcomes for chest injury patients. Chest x-ray remains to be an imaging modality for diagnosing thoracic trauma lesions.

### Recommendation

Preventive measures targeting reducing the occurrence of MTCs are necessary further to reduce the prevalence of chest injuries in this region.

Lower health centers should be given continuous training on trauma management especially chest trauma, and emphasize early referral.

Management protocol or guideline review is suggested to have better and early intervention.
